# The Impact of the Competition on miRNA, Proteins, and Metabolites in the Blood Exosomes of the *Yili* Horse

**DOI:** 10.3390/genes16020224

**Published:** 2025-02-15

**Authors:** Xinxin Yuan, Xinkui Yao, Yaqi Zeng, Jianwen Wang, Wanlu Ren, Tongliang Wang, Xueyan Li, Lipin Yang, Xixi Yang, Jun Meng

**Affiliations:** 1 College of Animal Science, Xinjiang Agricultural University, Urumqi 830052, China; xjauyxx@126.com (X.Y.); yxk61@126.com (X.Y.); xjauzengyaqi@163.com (Y.Z.); wjw1262022@126.com (J.W.); 13201295117@163.com (W.R.); wtl13639911402@163.com (T.W.); 13139883908@163.com (X.L.); yanglp98@126.com (L.Y.); xxyang2022@126.com (X.Y.); 2 Xinjiang Key Laboratory of Equine Breeding and Exercise Physiology, Urumqi 830052, China

**Keywords:** *Yili* horse, competition, extracellular vesicles, miRNAomics, proteomics, metabolomics

## Abstract

Purpose: Horse racing may cause stress-induced physiological changes and tissue damage in horses, but the changes in miRNA expression, protein expression, and metabolic substances in the plasma exosomes of the *Yili* horse after racing are still unclear. This study detected miRNA, protein expression, and metabolic substances in the plasma exosomes of *Yili* horses before and after competition, providing new insights for post-race recovery and care of *Yili* horses. Method: Eight three-year-old *Yili* horses that had undergone training were selected as the research subjects, with four horses that had not competed as the control group and four horses that had participated in the competition for half an hour as the training group. Extract whole blood and separate plasma from two groups of horses, and then extract plasma exosomes; MiRNAs, proteins, and metabolites in extracellular vesicles were detected and analyzed using miRNAomics, proteomics, and metabolomics. P Result: After the competition, the levels of miRNAs related to the cytoplasm and nucleus in *Yili* horse plasma exosomes increased, and miRNAs related to the transcription and transcriptional regulation of biological processes significantly increased. The levels of proteins related to the cytoplasm and nucleus also increased, and the levels of proteins related to cell signaling function increased, carbohydrates and their metabolites were significantly reduced. Conclusions: The competition process causes significant changes in the miRNA, proteomics, and metabolomics of plasma exosomes in the *Yili* horses, which are mainly related to metabolic regulation.

## 1. Introduction

Horse racing is a sport with significant economic and cultural value [1]; meanwhile, horse racing may cause muscle and bone damage to horses [2]. The medical care of horses after horse racing is an important research direction in horse medicine [3]. There are currently multiple invasive and non-invasive methods for treating injuries in horse racing, but further investigation into the mechanisms of injury is still needed to seek better treatment options [4]. Considering that different horses should be treated as specific individuals [5], it is particularly crucial to find individual specific biological indicators for personalized treatment.

Extracellular vesicles are biologically active vesicles secreted by cells that can carry miRNAs and proteins [6]. Research has found that extracellular vesicles have the ability to repair cell damage [7] and increase collagen accumulation and migration in horse joint chondrocytes [8], serving as potential biological indicators of physiological recovery ability [9]. Sports events stimulate the release of extracellular vesicles into the circulation [10], and extracellular vesicles can have a significant impact on the physiological functions of exercise [11]. Extracellular vesicles can serve as a medium for the body to adapt to endurance sports [12] and play a role in rebuilding damaged muscles [13].

MicroRNA (miRNA) are small non-coding RNA molecules [14] that can regulate gene expression post-transcriptionally and are present in body fluids such as plasma, saliva, and urine [15]. Exosomes can serve as transport carriers for miRNA [16] and can be used as biomarkers for disease diagnosis [17]. Studying the differences in exercise can cause changes in miRNA levels in human plasma, which can vary with the degree of exercise [18]. miRNA can serve as a potential biomarker for different types of exercise [19], such as a biomarker for changes in the endurance exercise response system [20], and has a protective effect on the body during exercise [21]. However, the changes in miRNAs in *Yili* horse plasma exosomes after competition are still unclear.

Proteomic analysis can be used to analyze various body-fluid-related data of horses [1]. For example, proteomic analysis shows that the estrous cycle causes significant differences in the protein expression of uterine wash fluid in horses [22], and has been found that the pregnancy status of horses causes differential expression of serum proteins [23]. Therefore, a quantitative analysis of the serum proteome in early pregnancy of horses can be carried out to analyze the protein expression mechanism related to horse pregnancy [24]. However, the impact of competition on *Yili* horse plasma exosome proteins is still unclear.

Blood metabolites change with changes in the body’s health status. For example, the metabolomics of mare follicular fluid are correlated with cyclic follicular changes [25], and changes in metabolic indicators can distinguish whether or not horses suffer from osteoarthritis [26]. Exercise can cause changes in plasma metabolites [27], inducing an increase in hemoglobin concentration and lactate in horse blood [28], suggesting that exercise may cause changes in horse plasma exosome metabolites. However, the impact of competition on *Yili* horse plasma exosome metabolites is still unclear.

A study found that endurance exercise alters the blood metabolomics, transcriptomics, and miRomics of horses [29]. We hypothesize that after horse racing, there will be significant changes in miRNA, proteins, and metabolites in horse plasma exosomes. Exploring these changes is of positive significance for exploring the health care and recovery of horses after horse racing. This study uses miRNA omics, proteomics, and metabolomics techniques to detect plasma exosomes in horses after horse racing in order to better understand the physiological changes in horses after horse racing and provide a reference and potential biomarkers for horse racing care.

## 2. Method

### 2.1. Experimental Animals and Grouping

In order to minimize the impact of environmental factors on horses as much as possible, this study screened the horses used in the study from the Yili Zhaosu Racecourse in the Yili Zhaosu area based on our inclusion criteria. Due to limitations in the scope of sample sources, the number of research subjects was restricted; this study used eight three-year-old male *Yili* horses as research subjects. All horses started training at the age of two and reached the level of being eligible to participate in the 2023 “Yueyang Tower Silk Road Cup” 5000 m speed race organized by the Chinese Jockey Club. All participating horses had not taken any medication, had no medical history, were in good health, and had passed doping tests. Four horses before participating in the race were used as the group before the competition (control group), and four horses within half an hour after participating in the 5000 m speed race were used as the after the competition group (experimental group). Blood was collected from the jugular vein; 20 mL of fresh blood was collected from each horse into a blood collection tube containing heparin sodium, and the plasma was separated by centrifugation at 3000 rpm for 5 min at 4 °C. Then, the plasma was separated and stored in liquid nitrogen for future use.

### 2.2. Extracellular Vesicle Extraction

This study used ultracentrifugation to extract extracellular vesicles. In general, horse plasma was separated and stored in liquid nitrogen. Prior to extraction, the plasma was thawed at 37 °C and centrifuged at 2000× *g* for 30 min at 4 °C. The supernatant was then transferred to a new centrifuge tube and centrifuged at 10,000× *g* for 45 min at 4 °C to remove larger vesicles. The supernatant was filtered using a 0.45 μm filter membrane, and the filtrate was centrifuged at 100,000× *g* for 70 min at 4 °C. Then, the supernatant was resuspended in 10 mL of pre-cooled 1×PBS and centrifuged at 100,000× *g* for 70 min at 4 °C to remove the supernatant. Then, the precipitate was resuspended in 200 μL of pre-cooled 1×PBS supernatant and store at −80 °C.

### 2.3. Transmission Electron Microscopy Observation

Hitachi’s HT-7700 projection electron microscope( Hitachi, Tokyo, Japan) was used for electron microscopy observation. An amount of 10 μL of extracellular vesicles was taken out, and 10 μL of the sample was dropped onto copper mesh for 1 min to precipitate. The float was removed by filter paper, and 10 μL of uranyl acetate was dropped onto the copper mesh for 1 min to precipitate. The float was removed by filter paper and dried at room temperature for several minutes. Then, electron microscopy imaging was performed at 100 kV to obtain transmission electron microscopy imaging results.

### 2.4. miRNA Sequencing

Using absorbance spectrophotometry for RNA sample quality testing, when the A260/A230 ratio of the sample was greater than 2 and the A260/A280 ratio was between 1.8 and 2.1, it was used for subsequent detection. If the RNA sample did not meet the above conditions, the RNA was extracted from the sample again, and only after meeting the above conditions was it used for library construction. The process of library construction strictly adheres to the guidelines provided by the NEB Next Ultra Small RNA Sample Library Prep Kit (New England Biolabs, Ipswich, MA, USA) for Illumina. Once the libraries were deemed qualified, they were then used for high-throughput sequencing. The sequencing was carried out on the Illumina HiSeq X Ten platform (New England Biolabs, Ipswich, MA, USA), with a read length of single-end (SE) 50 nucleotides.

### 2.5. Quantitative Proteomics

The total steps for differential quantitative proteomic analysis of extracellular vesicles using the diaPASEF acquisition mode of the timsTOF Pro2 series mass spectrometer in this study were as follows: adding 1 mM PMSF and 2 mM EDTA (final concentration) lysis buffer (8 M urea) to the sample for 5 min, sonicating for 5 min, centrifuging the lysis buffer at 4 °C and 15,000× *g* for 10 min, collecting the supernatant, and determining the total protein concentration through BCA protein quantitative analysis. An equal amount of the protein solution was taken according to the protein concentration, the volume was filled with 8 M urea to 200 μL, and then it was reduced with 10 mM DTT at 37 °C for 45 min and alkylate with 50 mM iodoacetamide (IAM) in a dark room at room temperature for 15 min. Four times the volume of pre-cooled acetone was added to the protein solution, and it was precipitated at −20 °C for 2 h. After centrifugation, the protein precipitate was air dried and resuspended in 200 μL of 25 mM bicarbonate solution and 3 μL of trypsin (Promega, Madison, WI, USA) and digested overnight at 37 °C. After digestion, the peptides of each sample were desalted on a C18 column, concentrated by vacuum centrifugation, and re-dissolved in 0.1% (*v*/*v*) formic acid for machine analysis.

### 2.6. Liquid Chromatography Tandem Mass Spectrometry (LC-MS/MS)

This study used liquid chromatography tandem mass spectrometry (Bruker, Billerica, MA, USA) for full-spectrum metabolomics detection. The total steps were as follows: extracellular vesicle samples were taken from a −80 °C refrigerator and thawed on ice (all subsequent operations required performing on ice); 500 μL of an 80% methanol water internal standard extraction agent was added, and the sample was vortexed for 3 min. Then, the centrifuge tube was frozen in liquid nitrogen for 5 min, thawed on ice for 5 min, thawed on ice for 5 min, vortexed for 2 min, and then repeatedly placed in liquid nitrogen, thawed, and vortexed 3 times. Under 4 °C centrifugation at 12,000 r/min for 10 min, 450 μL of the supernatant was transferred to a new centrifuge tube and concentrated to complete dryness; then, 100 μL of 70% methanol–water was added for reconstitution, and the sample was vortexed for 3 min and placed in ice water. The sample was ultrasonicated in the bath for 10 min and centrifuged at 12,000 r/min for 3 min at 4 °C; then, 80 μL of the supernatant was transferred into the corresponding injection bottle tube for machine analysis.

### 2.7. Bioinformatics Analysis and Statistical Analysis

Bioinformatics Analyses were performed using the following, accessed on 10 March 2024: Gene Ontology (GO) analysis using http://geneontology.org/, Cluster of Orthologous Groups of proteins (COG) using https://www.ncbi.nlm.nih.gov/research/cog-project/, and using Kyoto Encyclopedia of Genes and Genomes (KEGG) https://www.genome.jp/kegg/.

## 3. Result

### 3.1. The Competition Significantly Altered the Expression of miRNA in Horse Plasma Exosomes

**Figure 1:**
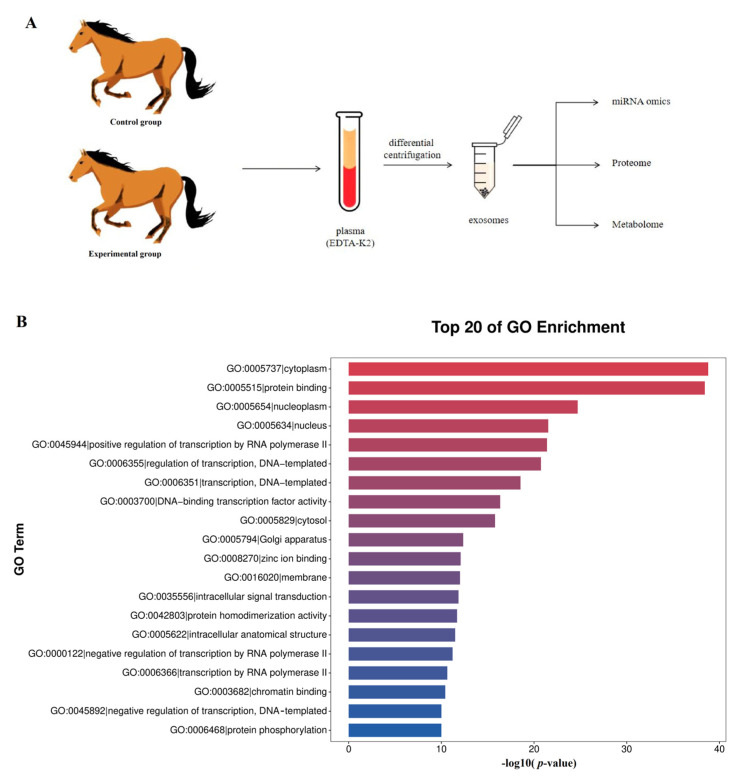
(**A**) Roadmap of this research: after collecting plasma from two groups of *Yili* horses, extracellular vesicles were isolated and subjected to miRNA omics, proteomics, and metabolomics detection. (**B**) Analysis of miRNA GO enriched cellular components for differential expression of plasma exosomes between the two groups: the vertical axis is an annotation of the cell components shown in each panel, and the horizontal axis displays the differences in different cell components.

**Figure 2:**
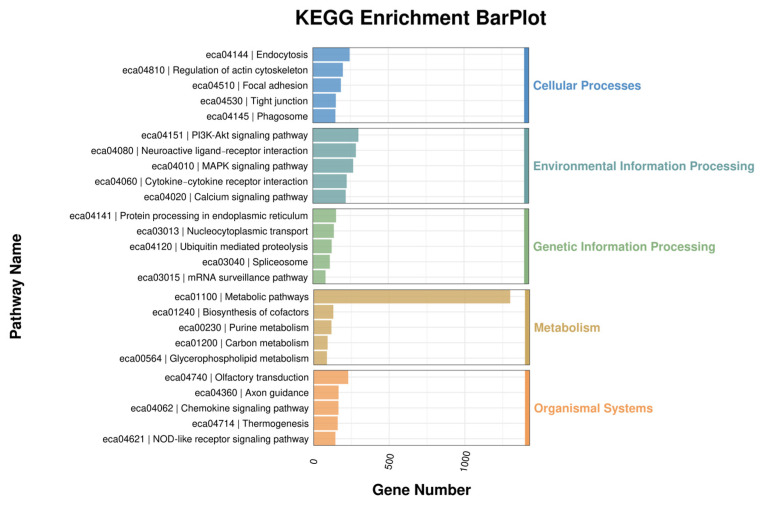
KEGG enrichment analysis chart. KEGG enrichment analysis was performed on the biological processes of differentially expressed miRNAs in horse plasma exosomes between the control group and the training group.

**Figure 3:**
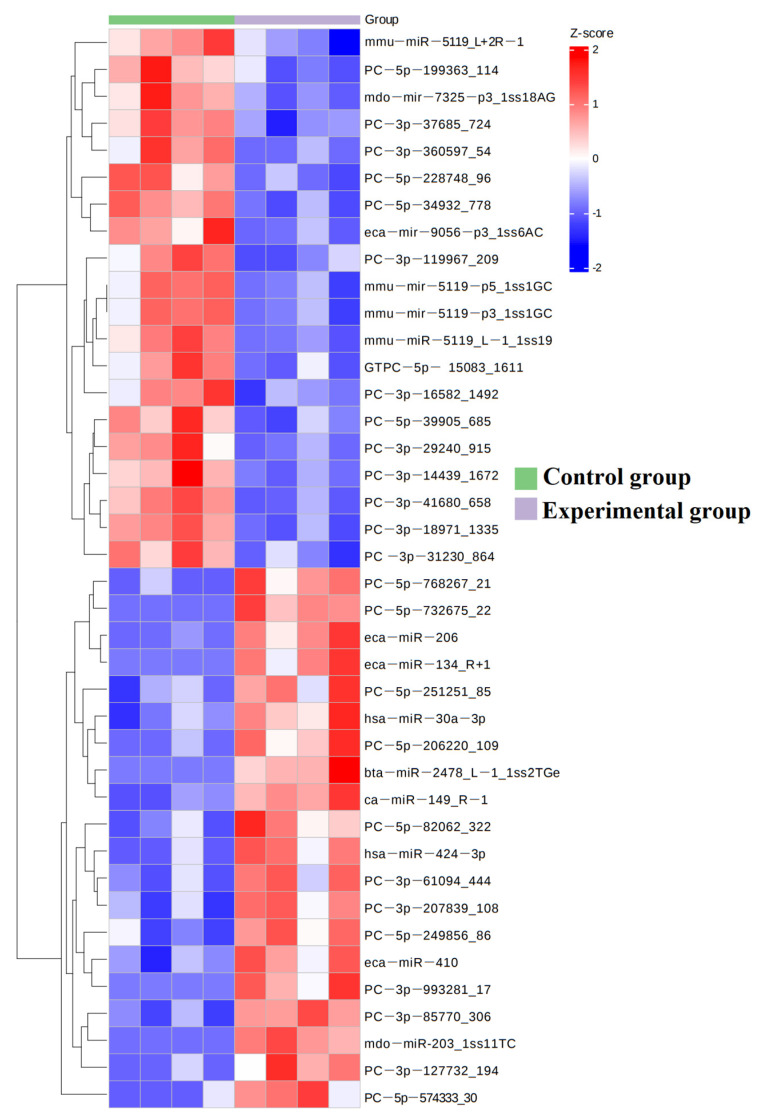
MiRNA composition heatmap, arranged in the order of sample grouping. In the figure, the red color block represents a higher abundance of the genus in this sample compared to other samples, while the blue color block represents a lower abundance of the miRNA in this sample compared to other samples.

The roadmap of this experiment is shown in Figure 1A. The collected exosomes were detected for miRNA by miRNAomics analysis. The GO enrichment analysis showed that the increase in miRNA in cell components related to the cytoplasm, protein binding, the nucleoplasm, and the nucleus was the most significant (Figure 1B). The KEGG analysis showed that miRNA related to metabolic pathways increased the most (Figure 2). In order to further compare the differences in species composition between samples and display the miRNA levels of each sample, a heatmap was drawn for the top 20 miRNAs with average increase and decrease differences (Figure 3).

### 3.2. The Competition Significantly Altered the Protein Expression of Horse Plasma Exosomes

**Figure 4:**
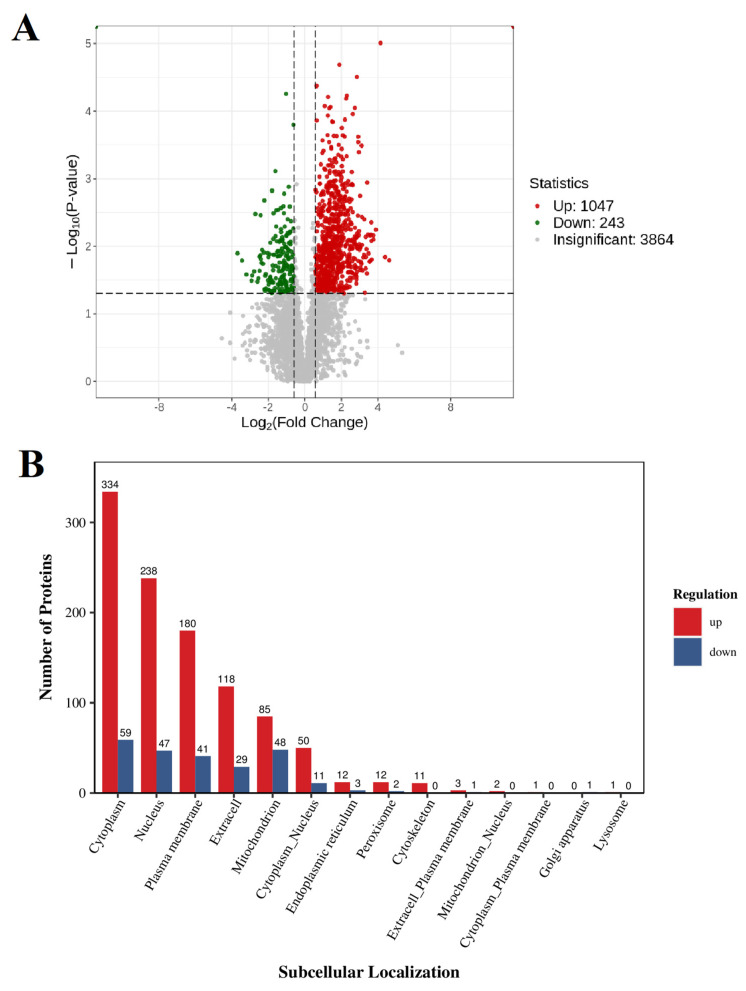
(**A**) Volcanic diagram of differential proteins in extracellular vesicles. The horizontal axis represents log2, the vertical axis represents the log10 *p* value, and the red and green scatter dots represent upregulated and downregulated differentially expressed proteins, respectively. The gray dots represent proteins with no significant differences. (**B**) A bar chart comparing the up- and downregulation of subcellular localization results, where the horizontal axis represents the subcellular region, the vertical axis represents the number of differentially expressed proteins annotated in that subcellular region, and the red and blue colors represent upregulated and downregulated differentially expressed proteins, respectively.

**Figure 5:**
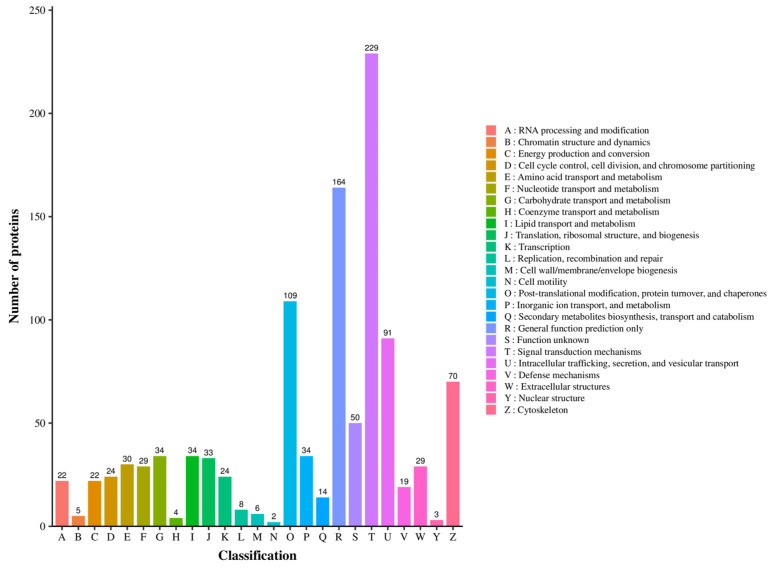
Cluster of orthologous groups of proteins bar chart. The horizontal axis represents the functional classification of KOG, the vertical axis represents the number of differentially expressed proteins annotated to corresponding functions, and the legend on the right represents the description of functional classification.

**Figure 6:**
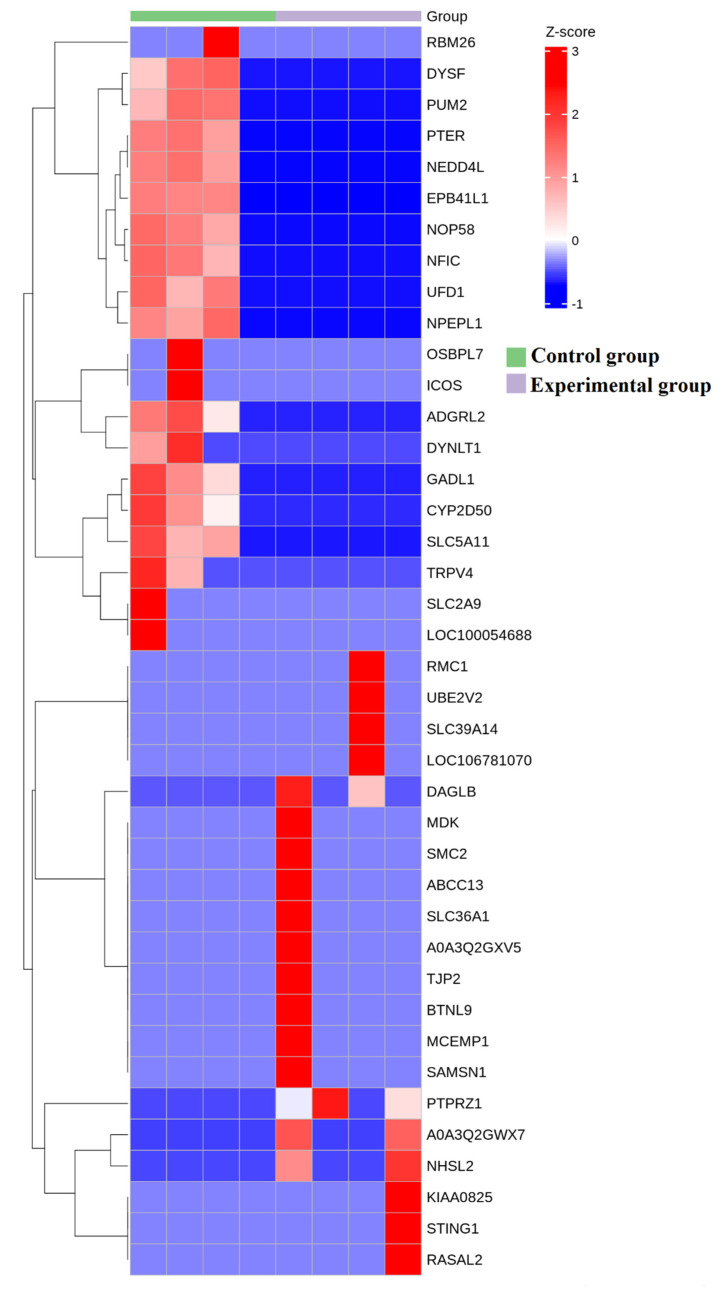
Protein composition heatmap, arranged in the order of sample grouping. In the figure, the red color block represents a higher abundance of the genus in this sample compared to other samples, while the blue color block represents a lower abundance of the proteins in this sample compared to other samples.

The volcano plot analysis showed that after the competition, 1047 proteins in the plasma exosomes significantly increased, 243 proteins significantly decreased, and 3864 proteins did not show significant changes (Figure 4A). However, according to the cellular components, the proteins with the highest increase were in the cell membrane, nucleus, and plasma membrane (Figure 4B). The KOG functional analysis showed that miRNAs related to signal transduction mechanisms increased the most (229/1047) (Figure 5). In order to further compare the differences in protein composition between samples and display the proteins levels of each sample, a heatmap was drawn for the top 20 proteins with average increase and decrease differences (Figure 6).

### 3.3. The Competition Significantly Altered the Composition of Metabolites in Horse Plasma Exosomes

**Figure 7:**
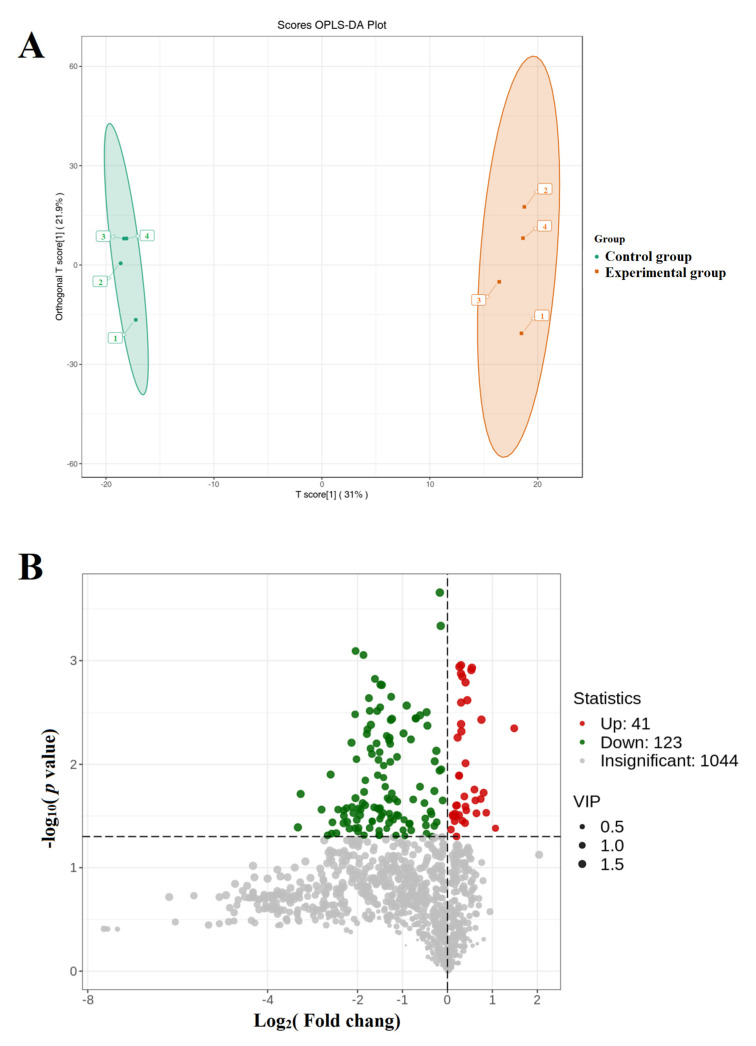
(**A**) OPLS-DA score chart. The horizontal axis represents the predicted principal components, and the difference between groups can be seen in the direction of the horizontal axis. The vertical axis represents the orthogonal principal components, and the difference within the group can be seen in the direction of the vertical axis. The percentage represents the explanatory power of the component on the dataset. Each point in the figure represents a sample, and samples in the same group are represented by the same color. Group represents grouping. (**B**) Volcanic diagram of differential proteins in extracellular vesicles. The horizontal axis represents log_2_, the vertical axis represents the log_l0_ *p*-value, and the red and green dots represent upregulated and downregulated differential proteins, respectively.

**Figure 8:**
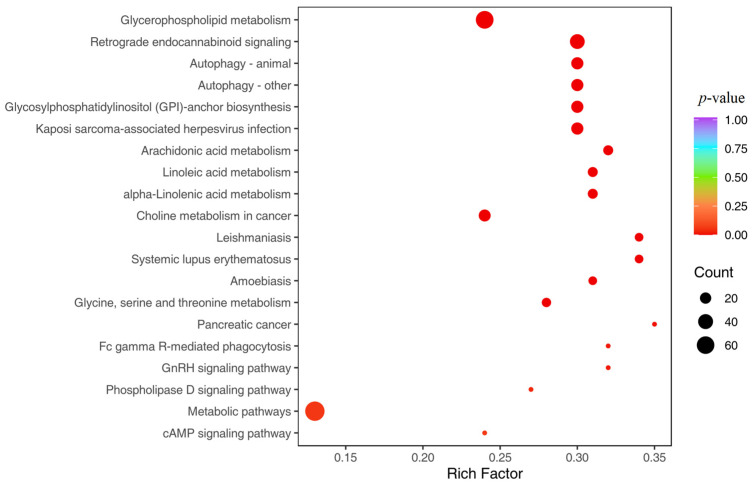
KOG enrichment analysis bubble chart. The horizontal axis represents the enrichment factor (DifRatio/BgLatio ratio), with a higher enrichment factor indicating a higher degree of enrichment of differentially expressed proteins, and the vertical axis represents the functional description of KOG. The color of the dots changes from blue to red, representing the change in *p*-value from large to small. The smaller the *p*-value, the more statistically significant it is. The size of the dots represents the number of differentially expressed proteins annotated with corresponding functions.

**Figure 9:**
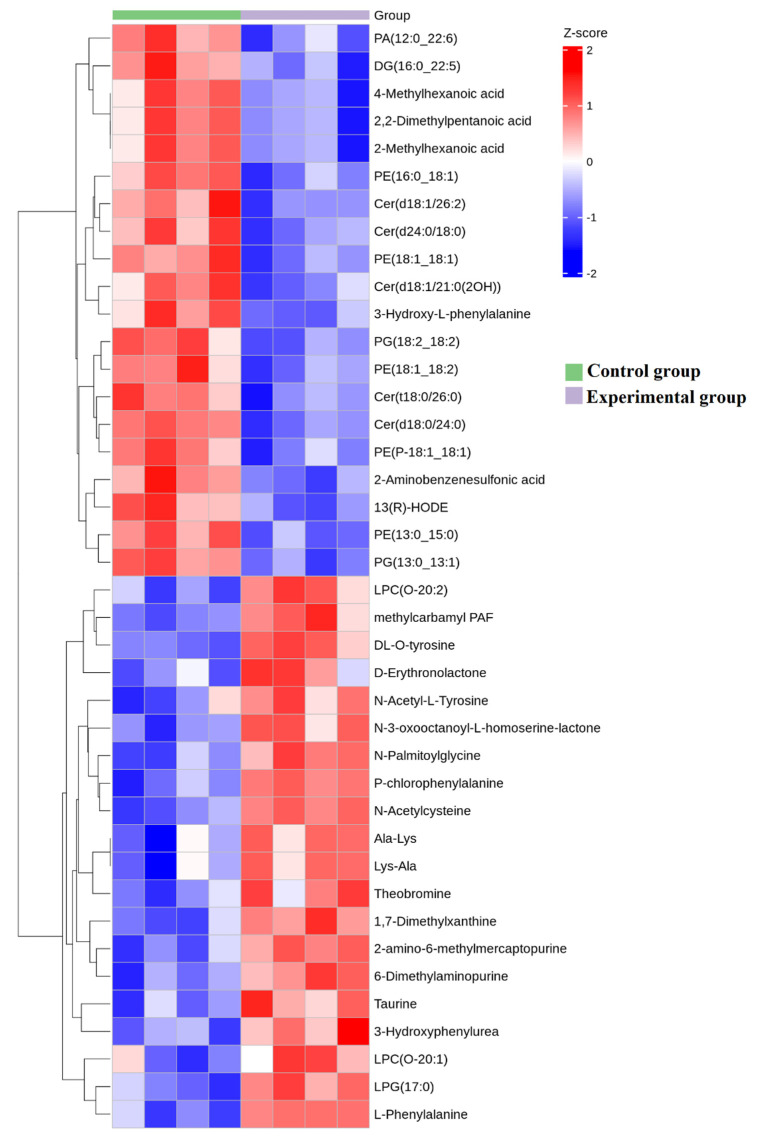
Metabolite composition heatmap, arranged in the order of sample grouping. In the figure, the red color block represents a higher abundance of the genus in this sample compared to other samples, while the blue color block represents a lower abundance of the metabolites in this sample compared to other samples.

Further detection of extracellular vesicle metabolites was conducted through metabolomics, and the OPLS-DA score analysis showed significant differences in the composition of extracellular vesicle metabolites after the competition (Figure 7A). The volcano plot analysis showed a significant increase in the expression of 41 metabolites and a significant decrease in the expression level of 123 metabolites in extracellular vesicles after the competition (Figure 7B). The KOG enrichment analysis revealed a significant increase in metabolites related to glycerophospholipid metabolism, retrograde endocannabinoid signaling, and autophagy-related biological pathways (Figure 8). In order to further compare the differences in species composition between samples and display the metabolite levels of each sample, a heatmap (Figure 9) was drawn for the top 20 metabolites with the highest and lowest average differences.

### 3.4. Joint Analysis of the Effects of Competition on Horse Plasma Exosome Metabolites and miRNA

**Figure 10:**
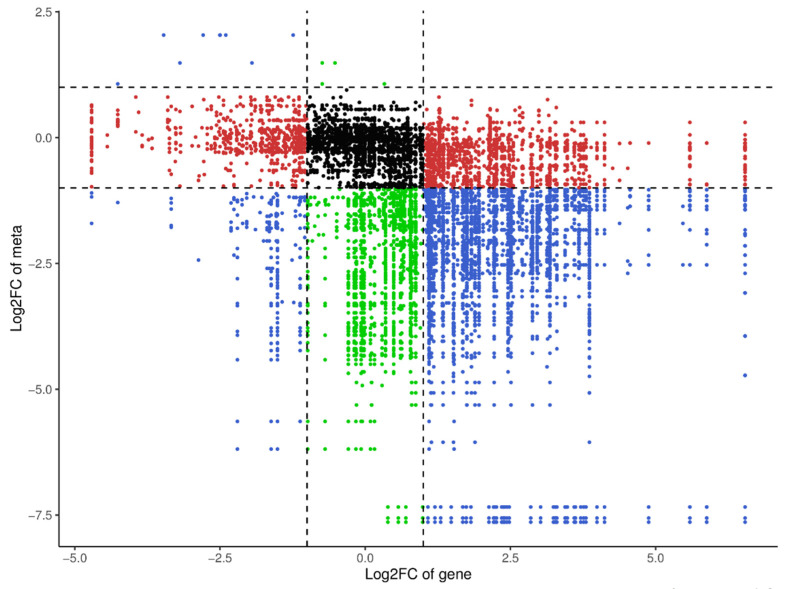
Correlation analysis: nine-quadrant chart in each differential group, selecting the correlation parts that meet the Pearson correlation coefficient with an absolute value greater than 0.8 and a *p*-value less than 0.05, where each point represents a pair of correlation relationships, the horizontal axis represents the Log2FC of the gene, and the vertical axis represents the Log2FC of the metabolite. It is divided into 1–9 quadrants using black dashed lines from left to right and from top to bottom, where miRNA and metabolites in quadrant 5 are not differentially expressed. The miRNAs in quadrants 3 and 7 have a positive correlation with metabolites, and the expression changes of metabolites may be positively regulated by miRNAs. The miRNAs in quadrants 1 and 9 have an inconsistent regulatory trend with metabolites, and the expression changes of metabolites may be negatively regulated by genes. The expression of metabolites in quadrants 2, 4, 6, and 8 remains unchanged, and miRNA is upregulated or downregulated, or miRNA expression remains unchanged, while metabolites are upregulated or downregulated.

**Figure 11:**
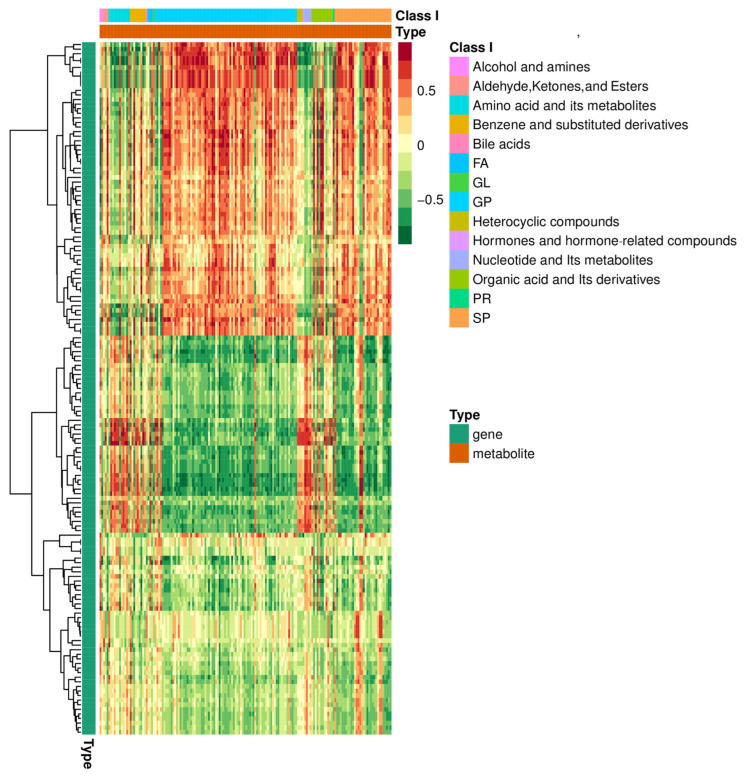
Correlation clustering heatmap, where each row represents a gene and each column represents a metabolite. Red represents a positive correlation between genes and metabolites, while green represents a negative correlation between genes and metabolites.

In order to reduce the impact of missing data and noise, we conducted a joint analysis of miRNA omics and metabolomics data to obtain more accurate conclusions. After the joint analysis of miRNAs and metabolites in two plasma groups, it was found that training significantly altered the expression of miRNAs and metabolites in the *Yili* horses’ plasma (Figure 10), with the most significant change observed being a significant increase in miRNAs related to fatty acids and sphingolipids during the competition (Figure 11).

## 4. Discussion

Intense exercise can damage the human body [30], which may involve damage to lipids, proteins, and nucleic acids [31]. Horse racing can also cause catastrophic musculoskeletal damage to horses [32]. The response of healthy subjects to acute aerobic exercise leads to an increase in plasma exosome abundance [33]. This study found that horse racing had a significant impact on miRNA, proteins, and metabolites in horse plasma exosomes.

There are studies showing that different miRNA subgroups are differentially expressed in a tissue-specific manner in horse equipment [34]. Among them, extracellular vesicles secreted by cells in horse follicular fluid contain miRNA and proteins that may be involved in cellular communication within ovarian follicles [35]. Pregnancy can affect miRNA expression in horse serum [36]. Muscle-specific miRNAs are regulated by human skeletal muscle endurance exercise [37]. Exercise can rapidly regulate the expression of miRNA in athletes’ skeletal muscle [38] and significantly alter the expression of miRNA in plasma exosomes [39]. These findings suggest that miRNAs have been found to be potential biomarkers for distinguishing the physiological status of horses [40]. MiRNAs can regulate endocytosis [41]. Through a KEGG enrichment analysis, this study found that the endocytosis process of miRNAs in plasma exosomes after horse racing was enhanced, which may be related to the transport of exosomes mediated by endocytosis [42] and the delivery of miRNAs [43]. The enhancement of the *MAPK* pathway also supports the speculation that the exercise-induced endocytosis pathway is enhanced [44]. The KEGG enrichment analysis also found that the metabolic pathways related to miRNA in horse plasma exosomes were most enhanced after the competition, suggesting that this is due to the increased cellular metabolic levels caused by exercise and the involvement of exosomal miRNA in metabolic regulation. The GO enrichment analysis showed a significant increase in miRNAs related to transcriptional regulation in biological processes, while there was an increase in miRNAs related to the cell membrane composition, cytoplasm, and nucleus, which is similar to the changes in miRNAs in skeletal muscle after exercise [38]. This may be related to the miRNA regulation of transcription [15]. The expression of hsa-miR-30a-3p is reduced in tumor tissues [45,46], and its downregulation is associated with inflammatory stimuli [47]. The expression level of hsa-miR-424-3p is upregulated in tumor tissues [48] and may be a prognostic marker [49]. This study showed that the levels of these two miRNAs increased in plasma exosomes after the competition, suggesting that they may not only play a role in tumor progression but also play a mediating role during exercise, but further research is needed to verify this.

Considering that the changes in muscle miRNA after exercise are influenced by gender [50], we believe that gender differences may lead to differences in miRNA expression in plasma exosomes after horse racing, which deserves further investigation.

There are studies showing that increased resistance to high-load exercise enhances hormone signaling [51]. This study found that the expression of extracellular vesicle proteins in *Yili* horse plasma changed after the competition. The GO enrichment analysis showed a significant increase in protein phosphorylation and signaling-related proteins, which may be related to the enhanced hormone signaling by increased resistance to high-load exercise [51]. The cellular components related to the cell membrane and nucleus significantly increased, and the protein binding to metal ions and calcium ions increased. Studies have shown that immune cells participate in the release of exosomes triggered by movement into the circulation [10]. Exosomes derived from endothelial progenitor cells promote anti-inflammatory macrophages and improve spinal cord injury through the *SOCS3*/*JAK2*/*STAT3* axis [52]. The KEGG analysis showed an increase in protein levels related to the transendothelial migration function of white blood cells, which may be related to the involvement of extracellular vesicles in the process of white blood cell metastasis [53]. At the same time, the levels of proteins related to endocytosis increased, which is consistent with the changes in miRNA. This may be related to the fact that exosomes can capture miRNA through endocytosis [54], and the internalization of exosomes occurs through endocytosis [55]. The protein function related to Th1 and Th2 cell differentiation is significantly enhanced, which may be due to the increase in cellular inflammation levels caused by the horse racing process, where exosomes regulate immune responses through exosome-related cytokines [56]. *DYSF* gene mutations lead to limb girdle muscular dystrophy [57], and *TRPV4* may be involved in the physiological protective function during hypoxic exercise [58], the data from this study show a decreasing trend in plasma exosomes, suggesting that muscle damage caused by horse racing may be related to changes in the expression of these two genes, and may be a potential therapeutic target. PUM2 protein is expressed in peripheral monocytes during horse racing [59], this study showed a decreasing trend in the expression of PUM2 in plasma exosomes during horse racing, which may serve as a biomarker for horse racing.

Exercise causes metabolic stress, and glycerophospholipids participate in metabolism [60] and can promote energy supply [61]. This study found that after horse racing, the levels of glycerophospholipids and sphingomyelins in the plasma exosomes of horses were significantly increased. Considering that exosomes can participate in signal pathway regulation [62], that the metabolism of phosphatidylcholine [63] and glycerophospholipids in exosomes is involved in signal pathway regulation, and that glycerophospholipid metabolism is involved in rheumatoid arthritis pathogenesis by regulating the IL-6/JAK signaling pathway, this may be related to the impact of exercise on the function of the sphingomyelin signaling pathway in skeletal muscle [64]. The KEGG analysis showed an increase in metabolites related to glycerophospholipid metabolism, which is consistent with differential changes in metabolites. The increase in metabolites related to retrograde endocannabinoid signaling may be related to synaptic excitation caused by exercise, and endocannabinoid retrograde signaling is involved in synaptic signaling [65]. At the same time, the increase in endocytic metabolites may be due to competition, enhancing mitochondrial energy metabolism and thereby enhancing endocytosis [66]. P-chlorophenylalanine may have antidepressant effects [67], and N-acetylcysteine has antioxidant functions [68] and can improve physical function [69] and participate in high-intensity exercise physiological processes [70]. This study showed that the levels of these two metabolites increased in plasma exosomes after competition, suggesting that competition causes horse plasma exosomes to participate in the transportation of corresponding metabolites to participate in the exercise process. Further research on their mechanisms may help to improve the exercise ability of horses in a targeted manner.

The development of omics technology provides opportunities for multi-omics testing [71]. Research has found that the plasma miRNA levels of endurance athletes and strength athletes are different [72], and the mode and degree of training can affect miRNA-based disease diagnosis [73]. Therefore, different levels of competition may cause different changes in plasma exosomes in *Yili* horses. Therefore, if exosomes are to be used as biomarkers for post-race diagnosis, it is necessary to explore the specific effects of competition on exosomes in order to better understand the impact of competition on exosomes and make accurate judgments.

## 5. Conclusions

This study found that the competition significantly altered the expression of miRNA, proteins, and metabolites in the plasma exosomes of *Yili* horses, providing new insights for post-care in *Yili* horses. However, the sample size of this study is relatively small, and future research needs to increase the sample size and expand the scope of the sampling site to increase the universality of data applicability and verify the regulatory mechanisms and physiological effects of its changes.

## Data Availability

The original contributions presented in this study are included in the article. Further inquiries can be directed to the corresponding author.
